# Special Issue “Challenges and Future Trends of Magnetic Sensors”

**DOI:** 10.3390/s25051307

**Published:** 2025-02-21

**Authors:** Galina V. Kurlyandskaya

**Affiliations:** Institute of Natural Sciences and Mathematics, Ural Federal University, Ekaterinburg 620002, Russia; galinakurlyandskaya@urfu.ru

In recent years, “sensor materials” have become a hot research topic in the field of applied research and industrial applications. Related works have appeared in many journals, and *Sensors* (MDPI) joined this list, responding to multiple requests. The list of particular topical advisory panel keywords truly reflects the multidisciplinary nature of this direction addressed to physicists, chemists, biologists, physicians, engineers for the large variety of problems that require solving, and even economists, psychologists, and specialists in educational problems.

Many traditional materials and composites are not suitable for the increasingly complex requirements of the fast-growing number of magnetic sensors and microsystems with magnetic components designed for automatization, navigation, industrial processes’ control, environmental control, biosensing and biomedical applications, drug delivery, and many others. The need for magnetic devices continues to challenge the materials science community to develop novel magnetic and composite materials that are suitable for such purposes. The principal requirements for a new generation of sensors are well known: high sensitivity, small size, low power consumption, stability, quick response, resistance to aggressive media, low price, and operated by non-skilled personnel. The increase in the number of nanomaterials available for research and applications requires that the methods of their characterization be even more precise than before. This Special Issue, entitled “Challenges and Future Trends of Magnetic Sensors”, intends to summarize the existing and new concepts related to material science, modelling, and technological achievements in the field of magnetic sensors in order to better understand the foreseeable future of these devices.

Around a year ago, we invited researchers to be an important part of this Special Issue, offering the following list of main keywords: magnetic effects; magnetic materials for sensor applications; magnetic field sensors; magnetic biosensors; hybrid sensors; modelling for magnetic sensor applications; complex structures; and composites for magnetic sensor applications. After thorough international reviewing, twelve contributions [Contributions 1–12] (one communication and eleven articles) were published. They represent international multidisciplinary teams from Brazil, China, Greece, Japan, Portugal, Russia, Spain, Turkey, and the United Kingdom.

Interestingly, different magnetic materials were the subject of the research published herein. Magnetic steels cover a large range of present-day applications, with transportation, energy, metallic buildings and bridges, and oil- and gas-related structures being vital for society in many manifestations; this topic was discussed by Hristoforou [Contribution 1]. Thin permalloy films and FeNi-based multilayered structures can be called two of the “eternal” subjects of magnetism, which is still receiving special attention [1,2]. In this Special Issue, a quantitative comparison of the model prediction and the measurement data for the microwave permeability of permalloy films was carefully analysed by Buznikov et al. [Contribution 2], indicating that changes in the magnetic structure of permalloy films appearing with an increase in film thickness are important. The observed decrease in the static permeability and frequency of the ferromagnetic resonance is related to the appearance of perpendicular magnetic anisotropy and the formation of stripe magnetic domains in the films.

The connection between the alternating current permeability and the structure of the magnetic domain was also analysed by Nakai [Contribution 9] for the case of amorphous Co_85_Nb_12_Zr_3_ films in the shape of magnetic stripes. The obtained results are promising for applications in the areas of tunable inductors, electromagnetic shielding, or magnetic sensing for the detection of the stray fields generated by magnetic nanoparticles.

Another example of the thin film sensitive element is given by Milyaev et al. [Contribution 10]. Spin valves, i.e., the structures consisting of two ferromagnetic layers, the free layer and the pinned layer, which are separated by a nonmagnetic spacer, are well-known materials in sensor applications [3,4]. It was shown [Contribution 10] that the fabricated rhombus-shaped CoFeNi/Ru/CoFeNi spin valves were synthetic antiferromagnets. The fabricated sensor elements in which each side of the rhombus was the shoulder of a Wheatstone bridge enabled the device to operate as a full Wheatstone bridge. The sensor had a high sensitivity to the external field changes, and significant magnetic hysteresis is suitable for switching devices.

Co-based amorphous ribbons were the subject of interest of the studies of Bukreev et al. [Contribution 11] and Correa et al. [Contribution 12]. In the first case, the authors compared the results of a computer simulation and experimental study of the magnetoimpedance effect (MI) in amorphous Co_68.5_Fe_4.0_Si_15.0_B_12.5_ and Co_68.6_Fe_3.9_Mo_3.0_Si_12.0_B_12.5_ ribbons, showing that the maximum MI value exceeds 200%. Such a high value may be of interest for the development of magnetic field sensors. In addition, it was shown that the practically significant characteristics of the MI response strongly depend on the driving current frequency due to the inhomogeneous distribution of magnetic properties over the cross-section. This distribution was studied using the magnetoimpedance tomography approach, also applied by Buznikov et al. [Contribution 7].

Correa et al. [Contribution 12] described thermoelectric phenomena, such as the Anomalous Nernst and longitudinal spin-Seebeck effects in Co-rich rapidly quenched ribbons and ribbon/Pt heterostructures, showing that Pt cover layer deposition leads to an enhancement of the thermoelectric response. The advantages of designing thermopiles consisting of Co-rich ribbon/Pt heterostructures in a parallel association were also shown. The longitudinal spin-Seebeck effect has been the subject of research for many years. For example, Uchida et al. [5] studied a longitudinal spin-Seebeck effect (SSE) in the case of ferrimagnetic insulator Y_3_Fe_5_O_12_, where the spin current was injected along the direction of a temperature gradient from a ferromagnet into a covering paramagnetic Pt layer. More recently, Ravi el al. [6] described this closely related research direction as flexible spin-caloritronic materials with thermoelectric conversion by nanostructure engineering.

Contributions 7–8 are devoted to the study of glass-coated magnetic microwires, which can be described as versatile magnetic composites with a large variety of ways to tune the response of a magnetic sensitive element adapting a microdevice to the particular needs of the application [7,8].

Zhang et al. [Contribution 4] described a two-point magnetic gradient localization method for remote, single-magnetic dipole targets based on SQUID magnetometers. A linear localization model based on the spatial position relationship between a magnetic moment vector and relative position vectors—which allow for the realization of the high-precision localization of a magnetic target and the calculation of its magnetic moment vector—was used. The simulations data and experimental results demonstrated very high localization performance for remote magnetic targets.

Although printed electronics have been used for decades, improvements in the corresponding fabrication techniques have recently gained a lot of attention when it comes to printing smart magnetic materials for sensors and actuators [9,10]. In the present Special Issue, the printed electronics direction was represented by Ahmed et al. [Contribution 6], who described the advantages of the inkjet printing of magnetostrictive materials for structural health monitoring in the case of carbon fibre-reinforced polymer composites. For example, it was shown how the change in the design and number of layers affects the value of inductance under a particular applied strain. 

Barrera et al. [Contribution 5] directed our focus to the hot research topic related to the biomedical applications of magnetic materials, in particular to the problem of the detection of magnetic nanoparticles in a liquid medium and the quantification of their concentration using the magnetoimpedance effect (MI). MI sensors have attracted much attention due to their high sensitivity to the stray magnetic field generated by magnetic nanoparticles, their simple fabrication process, and their relatively low cost [5,11,12]. In Contribution 5, the authors described an MI sensor with an amorphous Fe_73.5_Nb_3_Cu_1_Si_13.5_B_9_ wire-sensitive element integrated into the millifluidic chip to detect the presence of magnetic nanoparticles of the stabilized magnetic suspension by the quantification of stray fields generated by single-domain superparamagnetic iron oxide or magnetically blocked Co-ferrite nanoparticles. In fact, directly or indirectly, not only Contribution 5 but also Contributions 2, 3, 7, 8 and 11 touch on issues related to the dynamic properties of magnetic materials and MI. In 2024, it was the 30-year anniversary of the discovery of MI, i.e., the change in the complex impedance *Z* under the application of a constant magnetic field [13,14,15]. *Z* depends on the frequency *f* and amplitude *I*_ac_ of the alternating current flowing through the ferromagnetic conductor. The effect is understood in the frame of the classic electrodynamics interpretation through the connection of a change in the skin depth and magnetic permeability of a soft ferromagnet being very close to the L.D. Landau approach [16].

The year 2024 also marks 30 years since the re-discovery of the magnetoimpedance effect. Variation in the complex impedance *Z*, under the application of a constant external magnetic field *H*, is called magnetoimpedance (MI). Complex impedance *Z* depends on the frequency and amplitude of the alternating driving current flowing through the ferromagnetic conductor. The phenomenon of the dependence of the alternating current (ac) resistance of cold drawn iron–nickel wires on the value of the applied constant magnetic field was reported by the authors of [17,18]. They proposed a classic electrodynamics interpretation of the observed effect through the connection of a change in the skin depth and magnetic permeability of a soft ferromagnet being very close to the L.D. Landau approach [16]. However, the existence of some poorly controlled technological parameters caused a large variation in the dynamic magnetic properties, even in the cold-drawn wires of the same batch. Therefore, for about six decades, the resistance of variations in the ferromagnetic conductor under the application of a magnetic field has not attracted the attention of many researchers. Later, new soft magnetic materials with stable properties appeared, and fabrication techniques were significantly improved. In 1991, Makhotkin et al. [13] published results related to a low-magnetic-field sensor operating with an FeCoSiB amorphous ribbon-sensitive element describing the functionality principle as a change in the electrical impedance of the ribbon under the application of the magnetic field. In 1994, a set of publications related to MI appeared, and the terms giant magnetoimpedance and magnetoimpedance become well established [14,15]. Thirty years later, we can see that MI sensors have extraordinary sensitivity with respect to the applied magnetic field [11,19], are they are useful for operations control, current and position sensing, intelligent system monitoring, gradient field detection, non-destructive and automobile control, as well as for bio-magnetic measurements at room temperature and other biomedical applications [Contribution 5].

The year 2024 also marks the experimental confirmation of the idea of “altermagnetism”, an emerging magnetic phase characterized by robust time-reversal symmetry breaking. In this type of magnetic, it was noticed that materials with mixed properties were different from other types of materials of the magnetic phenomenon. Similarly to antiferromagnets, electrons in altermagnets spin in alternating directions and do not producing magnetization. However, the energy bands also have alternating spins from neighbouring bands. Altermagnetism is now considered the third elementary type of magnetic phases, in addition to the conventional ferromagnets and antiferromagnets [20]. According to experts’ opinions, altermagnets have the capacity to revolutionize spintronics, data storage, and magnetic sensing devices, providing enhanced efficiency and durability in comparison with traditional ferro- and antiferromagnets [20].

We expect this Special Issue and its corresponding book to be useful for graduate and PHD students, researchers working in the field of magnetic materials and nanocomposites and electronic engineering, and even personnel connected with biomedical applications.
